# The La protein counteracts cisplatin-induced cell death by stimulating protein synthesis of anti-apoptotic factor Bcl2

**DOI:** 10.18632/oncotarget.8819

**Published:** 2016-04-18

**Authors:** Tilman Heise, Venkatesh Kota, Alexander Brock, Amanda B. Morris, Reycel M. Rodriguez, Avery W. Zierk, Philip H. Howe, Gunhild Sommer

**Affiliations:** ^1^ Medical University of South Carolina (MUSC), Department of Biochemistry & Molecular Biology, Charleston, SC 29425, USA

**Keywords:** La/SSB, LARP3, RNA-binding protein, cisplatin, mRNA translation

## Abstract

Up-regulation of anti-apoptotic factors is a critical mechanism of cancer cell resistance and often counteracts the success of chemotherapeutic treatment. Herein, we identified the cancer-associated RNA-binding protein La as novel factor contributing to cisplatin resistance. Our data demonstrate that depletion of the RNA-binding protein La in head and neck squamous cell carcinoma cells (HNSCC) increases the sensitivity toward cisplatin-induced cell death paralleled by reduced expression of the anti-apoptotic factor Bcl2. Furthermore, it is shown that transient expression of Bcl2 in La-depleted cells protects against cisplatin-induced cell death. By dissecting the underlying mechanism we report herein, that the La protein is required for Bcl2 protein synthesis in cisplatin-treated cells. The RNA chaperone La binds in close proximity to the authentic translation start site and unwinds a secondary structure embedding the authentic AUG. Altogether, our data support a novel model, whereby cancer-associated La protein contributes to cisplatin resistance by stimulating the translation of anti-apoptotic factor Bcl2 in HNSCC cells.

## INTRODUCTION

The human La protein (LARP3, La/SSB) is an RNA-binding protein that is overexpressed in various types of cancer [1–3], and stimulates proliferation, migration, and invasion of cancer cells *in vitro* [2–4] as well as tumor growth *in vivo* [5]. However, the underlying mechanism how the La protein contributes to cancer pathogenesis remains elusive. The ubiquitously expressed and predominantly nuclear La protein is well known to be involved in various steps of the RNA metabolism [6, 7], including processing of small non-coding RNAs like tRNA and miRNA precursors [8, 9]. Interestingly, the La protein stimulates translation of mRNAs that have structural RNA elements located within the 5′-untranslated region (5'UTR) in common, like stem-loop structures as described in Mdm2 [1], a negative regulator of tumor suppressor p53, or internal ribosomal entry sites (IRES) as described for various viral and cellular mRNAs including tumor-promoting factors such as X-linked inhibitor of apoptosis (XIAP), cyclin D1, and Laminin B1 [2, 4, 5, 10–18]. Furthermore, evidence is growing that La acts as an RNA chaperone during mRNA translation suggesting that La stimulates protein synthesis of specific target mRNAs by smoothening out structural obstacles located in 5′UTRs to enable ribosome scanning and start site recognition [15, 19–23].

Cisplatin is a chemotherapeutic agent widely used as an effective agent in the treatment of various types of malignant tumors, including head and neck squamous cell carcinoma (HNSCC) [24, 25]. However, clinical data link overexpression of anti-apoptotic factors such as B-cell leukemia/lymphoma 2 (Bcl2) with a poor prognosis and several inhibitors of anti-apoptotic factors are tested in clinical trials to overcome cisplatin resistance [24, 26–30]. In recent years ongoing efforts have been made to identify novel inhibitors targeting Bcl2 expression in cancer cells to improve the outcome of anticancer therapy [31, 32].

Our study suggests that overexpression of cancer-associated La contributes to resistance of HNSCC cells toward cisplatin-induced cell death. La depletion by siRNA-mediated knock down or transient expression of a dominant negative La mutant, which has repeatedly been applied to inhibit La-dependent translation of viral and cellular mRNA targets [1, 11, 12, 33], correlates with reduced Bcl2 expression and increased cisplatin sensitivity. *In vitro* and cell-based assays suggest that the La protein binds in close proximity to the translation start site of Bcl2 and stimulates mRNA translation by unwinding a secondary structure embedding the authentic translation start site.

## RESULTS

### La protein expression correlates with protein level of anti-apoptotic factor Bcl2 and cisplatin sensitivity

**Figure 1:**
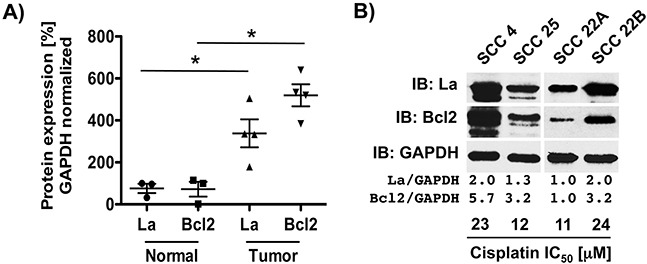
La protein level correlate with Bcl2 protein expression HNSCC cells **A.** Higher expression of La and Bcl2 protein in HNSCC tumor compared to normal tongue tissue lysates defined by immunoblot quantification and GAPDH normalization. **B.** In HNSCC cell lines high La protein level correlate with high Bcl2 protein expression and reduced sensitivity to cisplatin. Half maximal inhibitory concentration (IC_50_) of cisplatin was determined for each cell line.

We asked whether head and neck cancer-associated overexpression of La [3] counteracts cisplatin-induced apoptosis and thereby contributes to cisplatin resistance, which is often seriously impairing the therapeutic success in HNSCC patients. Since clinical data link expression levels of anti-apoptotic factor Bcl2 with cisplatin resistance and recurrent disease [24], we compared the expression of La and Bcl2 protein in tissue lysates obtained from normal tongue and tumor tissues of HNSCC patients. Immunoblot analysis showed that elevated expression of La protein correlates with increased Bcl2 protein expression in four HNSCC tumor tissues lysates when compared to three normal tongue tissue lysates (Figure 1A). Furthermore, we asked whether the cisplatin sensitivity of HNSCC cell lines correlates with La and Bcl2 protein level. Comparing four different HNSCC cell lines by immunoblot analysis our data show that low La protein expression correlates with low Bcl2 protein expression and high cisplatin sensitivity (low IC_50_ values: 12+/−1.9 μM in SCC 25 and 11+/−1.9 μM in SCC 22A) (Figure 1B, Supplementary Figure S1A and S1D). In contrast, high La expression correlates with high Bcl2 protein and low cisplatin sensitivity (high cisplatin IC_50_ values: 23+/−2.8 μM in SCC 4 and 24+/−2.7 μM in SCC 22B cells) (Figure 1B, Supplementary Figure S1B and S1C). Taken together, these data suggest a correlation between La, Bcl2 and cisplatin sensitivity in HNSCC cells.

**Figure 2:**
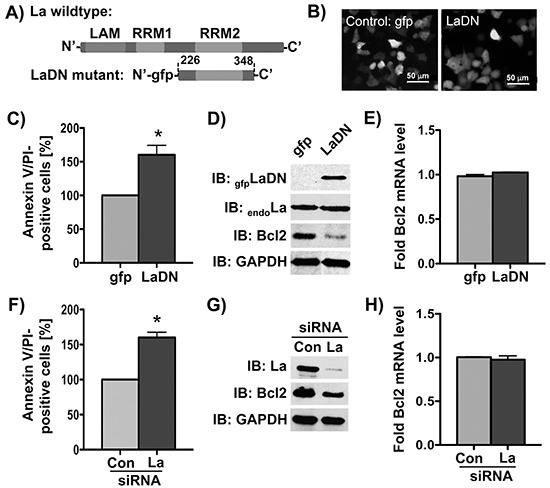
Bcl2 protein expression is reduced by siRNA-mediated La depletion or transient expression of La dominant negative (LaDN) mutant in cells **A.** Scheme of LaDN mutant compared to La wildtype containing three RNA-binding motifs: LAM, RRM1, and RRM2. **B.** Fluorescence microscopic image of gfp or LaDN mutant expression in living SCC 22B cells. La depletion in SCC 22B cells by **C-E.** transient expression of La dominant negative (LaDN) mutant or **F-H.** La-specific siRNA (La siRNA), results in increased cisplatin-induced apoptosis (Annexin/PI-positive cells) after cisplatin treatment with 24 μM for 24 hours, reduced Bcl2 protein expression (GAPDH = loading control), and unchanged Bcl2 mRNA level as determined by RT-qPCR analysis and normalized to GAPDH mRNA. Con = control siRNA. *P* value < 0.05 (one asterisks). Data shown are representative of three independent experiments (n = 3).

Next we tested whether reduction of La protein expression will increase the sensitivity toward cisplatin in HNSCC cells. To reduce the La expression we applied two independent approaches: (1) siRNA- or shRNA-mediated depletion of La, and (2) transient expression of a known La dominant negative (LaDN) mutant. The LaDN mutant encodes a 13.5 kDa protein (Figure 2A) and carries a green fluorescence (gfp) tag at the amino-terminus, which enables us to monitor its expression in living cells by fluorescence microscopy (Figure 2B). Transient expression of LaDN has been shown to reduce La-stimulated mRNA translation in cells, which may be caused by LaDN interfering with homodimerization of the La protein [11, 33]. To test whether decreased La expression sensitizes cells for cisplatin, we experimentally reduced the La expression and determined the number of apoptotic cells (Annexin V/PI-positive) after cisplatin treatment (at IC_50_) by FACS analysis. Our data show that after cisplatin treatment the numbers of apoptotic cells (Annexin V/PI-positive) were significantly increased in cells transiently expressing the LaDN mutant (Figure 2C) or in siRNA-mediated La-depleted cells (Figure 2F), when compared to controls. We conclude that reduced La protein expression increases the sensitivity of SCC 22B cells toward cisplatin treatment.

**Figure 3:**
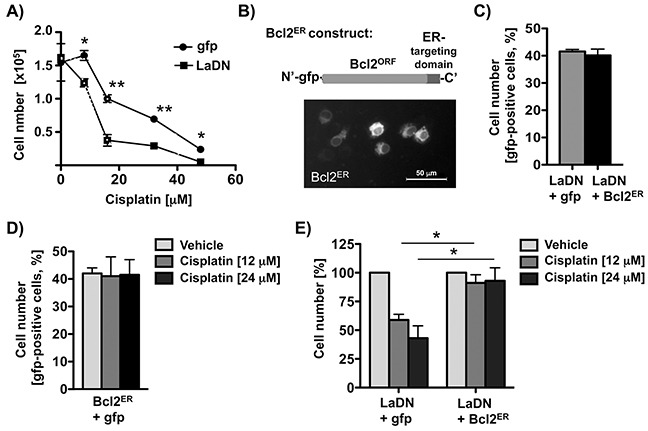
Transient expression of Bcl2^ER^ rescues cisplatin resistance of LaDN-expressing cells **A.** Cisplatin sensitivity is increased in LaDN- compared to gfp-expressing SCC 22B cells. Two independent experiments in triplicates were performed (*P* value < 0.05 (one asterisks), < 0.005 (two asterisks), n = 2). **B.** Upper panel: Scheme of Bcl2^ER^ construct. Bcl2's carboxy-terminal targeting sequence is replaced with Cytochrome b5, which targets Bcl2 to the endoplasmic reticulum (ER). Lower panel: Fluorescence microscopic image of living SCC 22B cells transiently expressing Bcl2^ER^. **C.** Control: Cell numbers are unchanged in cells expressing LaDN mutant alone or with Bcl2^ER^. **D.** Control: Cell numbers are unchanged in cells expressing Bcl2^ER^ alone, and treated with or without cisplatin. **E.** Increased cisplatin sensitivity of SCC 22B cells transiently expressing LaDN mutant can be rescued by co-expression of Bcl2^ER^. *P* value < 0.05 (one asterisks, n = 3).

Since the anti-apoptotic factor Bcl2 is well known to counteract cisplatin-induced apoptosis in cancer cells [24], its expression can be regulated by IRES-dependent translation [34], and because we observed a correlation between La and Bcl2 levels in HNSCC cells (Figure 1), we tested whether depletion of the La protein affects Bcl2 protein level. Our data demonstrate that Bcl2 protein expression is significantly reduced in cells that are La-depleted by either transient expression of mutant LaDN protein (Figure 2D), transfection of La-specific siRNA, or transduction with La-specific shRNA expression vectors (Figure 2G and 7A). Interestingly, reverse transcription (RT) followed by quantitative PCR (qPCR) analysis revealed unchanged Bcl2 mRNA levels (Figures 2E, 2H, and 7B) strongly suggesting that La stimulates Bcl2 protein expression post-transcriptionally. The correlation between La depletion, reduced Bcl2 protein expression and increased cisplatin sensitivity was confirmed in an independent cell line, SCC 22A (data not shown). To exclude off-target effects of La-specific siRNAs, we show that reduced Bcl2 protein expression in La-depleted SCC 22B cells is rescued by overexpression of La protein resistant to siRNA-mediated La depletion [2] (LaR, Supplementary Figure S2A). Furthermore, we demonstrate by two independent shRNAs that depletion of La reduces Bcl2 expression as well as X-linked inhibitor of apoptosis (XIAP), which is an anti-apoptotic protein earlier suggested to be translationally regulated by La [12] (Supplementary Figure S2B). To validate the link between La depletion and increased cisplatin sensitivity we transiently expressed gfp-tagged LaDN mutant or gfp as control. Our data reveal that the sensitivity toward cisplatin is significantly increased in LaDN-expressing cells compared to gfp-expressing control cells (Figure 3A).

Our next goal was to demonstrate that transiently overexpressed endoplasmatic reticulum (ER)-targeted Bcl2 (Bcl2^ER^, Figure 3B) could protect LaDN-expressing SCC 22B cells toward cisplatin treatment. Previous studies have shown that transient expression of Bcl2^ER^ mutant protects against cell death [35], whereas transient expression of wildtye Bcl2 induces cell death [35–37]. We transiently expressed Bcl2^ER^ mutant in SCC 22B cells either co-expressing gfp-tagged LaDN or gfp, treated with 12 or 24 μM cisplatin and determined the number of gfp-positive cells after 24 hours by FACS analysis. Strikingly, expression of Bcl2^ER^ in LaDN-expressing SCC 22B cells significantly rescued cisplatin-induced cell death (Figure 3E). Control samples of untreated cells co-expressing LaDN, gfp or Bcl2^ER^ (Figure 3C), or Bcl2^ER^ alone expressing cells treated with cisplatin (Figure 3D) did not show any reduced cell numbers. Taken together, these data demonstrate that transient expression of anti-apoptotic factor Bcl2^ER^ reduces the cisplatin sensitivity of LaDN-expressing cells.

### The La protein binds to a region of Bcl2 mRNA embedding the authentic translation start site

**Figure 4:**
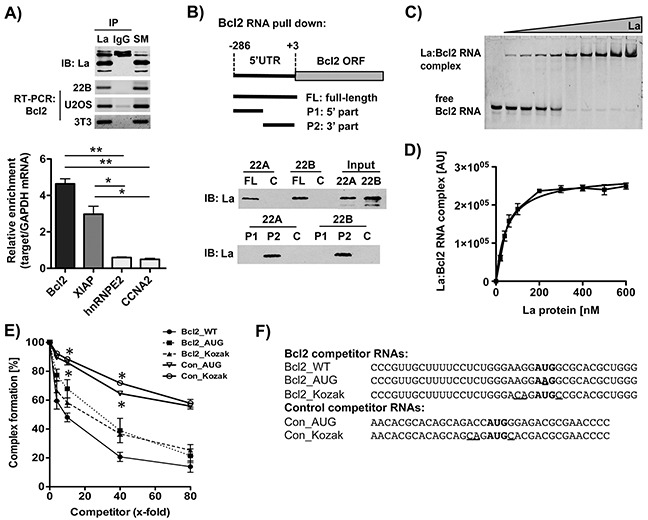
The La protein binds to a region of Bcl2 mRNA embedding the authentic translation start site **A.** Upper panel: RNA immuoprecipitation (RIP) applying a La-specific antibody followed by Bcl2-specific RT-PCR in three different cell lines (immunoblot (IB)). Lower panel: RIP followed by RT-qPCR analysis was performed in triplicates on RNA extracted from RIP pellets. Cell extracts for RIP experiments were prepared from HEK 293 cells stably transfected with gfp alone (control) or gfp-tagged La. None of the target mRNAs analyzed were detected in cells expressing gfp alone. Relative enrichment of target mRNA compared to GAPDH mRNA was calculated (n = 3). **B.** Scheme of Bcl2 RNA probes (FL, P1, P2) applied for RNA pull-down assays, and La-specific immunoblot (IB) following Bcl2 RNA pull down from SCC 22A and SCC 22B cell lysates. Data shown are representative of three independent experiments (n = 3). No RNA probe was added in the control (C). **C.** Binding affinity of La:Bcl2 RNA oligonucleotide interaction as determined by electrophoretic mobility shift assay (EMSA). The 5′FAM-labeled Bcl2 RNA oligonucleotide (25 nM) was incubated with increasing amounts of recombinant La protein in a range from 0 to 600 nM and separated by a native EMSA. **D.** The La:RNA complex formation is plotted against the La protein concentration. The dissociation constant (K_D_) was determined as 50.5+/−5.9 nM (n = 3) in Prism 5 (GraphPad Software). **E.** Competitive fluorescence polarization assay using 5′FAM-labeled Bcl2 RNA oligonucleotides and different unlabeled competitor RNA oligonucleotides at 4-, 10-, 40-, or 80-fold excess. *P* value < 0.05 (one asterisks), n = 3. **F.** Sequence of competitor Bcl2 RNA oligonucleotides (Bcl2_WT, Bcl2_AUG, Bcl2_Kozak) and two unrelated control RNA oligonucleotides (Con_AUG, Con_Kozak). Translation start site (AUG) in bold and nucleotides changed compared to wildtype (WT) RNA are underlined.

To reveal the mechanism whereby the RNA-binding protein La contributes to Bcl2 expression, we first performed RNA immunoprecipitation (RIP) experiments and demonstrated that La interacts with Bcl2 mRNA in three different cell lines (SCC 22B, U2OS, 3T3) (Figure 4A, upper panel). To further confirm the La:Bcl2 mRNA interaction, we established stable gfp or gfp-tagged La (_gfp_La) Introduce: -expressing HEK 293 cells and performed RIP experiments applying gfp-antibodies coupled to magnetic beads. RNA bound to _gfp_La or gfp alone was prepared, RT-qPCR performed and the relative enrichment of the target mRNA to GAPDH mRNA was calculated. Control experiments revealed that none of the target mRNAs tested bind to gfp alone demonstrating stringent washing conditions. Interestingly, our data indicate that Bcl2 mRNA binds to the La protein even better than XIAP mRNA, which was used as positive control (Figure 4A, lower panel). In contrast, negative controls hnRNPE2 and CCNA2 mRNA only showed minimal binding to the La protein in our RIP experiments (Figure 4A, lower panel).

Since it is known that La binds to the 5′UTRs of specific mRNAs and modulates their translation, we tested whether La binds to the Bcl2 5′UTR. Therefore, we performed RNA pull-down assays applying a biotinylated RNA probe spanning a region of the 5′UTR including the translation start site (nucleotides −286 to +3, Figure 4B, full-length (FL) Bcl2 RNA probe). Our data demonstrate that the FL-Bcl2 RNA probe efficiently pulled down La from SCC 22A and SCC 22B cell lysates (Figure 4B). To further narrow down the binding site of La within the Bcl2 5′UTR, the FL-Bcl2 RNA probe was divided in half. Pull-down experiments with P1- (nucleotides −286 to −126) and P2- (nucleotides −125 to +3) Bcl2 RNA probes indicate that La interacts with a region spanning 125 nucleotides upstream of and including the translation start site of Bcl2 mRNA (Figure 4B). Recent work shows that La binds the hepatitis C virus (HCV) and cyclin D1 5′UTR in close proximity of the translation start site [19, 21, 38]. To test whether La binds also near the Bcl2 translation start site a synthetic 5′-fluorophore (FAM fluorescein) labeled Bcl2 RNA oligonucleotide was synthesized. Electrophoretic mobility shift assays (EMSAs) demonstrate that increasing concentration of recombinant La leads to the formation of a La:Bcl2 RNA complex (Figure 4C) with an RNA-binding affinity of 50.5+/−5.9 nM (Figure 4D). These data were confirmed by La-based fluorescence polarization (La-FP) assays, an alternative quantitative method to monitor protein:RNA interactions (K_D_: 60.1+/−4.5 nM, Supplementary Figure S3A). Taken together these data demonstrate a robust interaction between La protein and Bcl2 mRNA in cells and *in vitro*.

To identify critical nucleotides in Bcl2 mRNA required for La binding, we applied competitive fluorescence polarization (cFP) assays using 5′FAM-labeled Bcl2 RNA oligonucleotides and several unlabeled competitor RNA molecules. As expected, increasing concentration of unlabeled Bcl2 RNA wildtype sequence (Bcl2_WT) efficiently competed for La:Bcl2 RNA complex formation (Figure 4E). In contrast, competitor RNA molecules with mutations in the translation start site (Bcl2_AUG) or the Kozak sequence (Bcl2_Kozak) were weak competitors, suggesting that the authentic translation start site embedded in a strong Kozak sequence contributes to some extend to binding of La (Figures 4E and 4F). To demonstrate specific binding of La to the Bcl2 mRNA, we tested whether two unrelated control_RNA oligonucleotides (Con_AUG, Con_Kozak, renamed from [39]) compete for binding of La protein to the Bcl2 mRNA. Both Con_AUG and Con_Kozak RNAs competed only minimal for La:Bcl2 RNA complex formation (Figure 4E), demonstrating that the AUG as well as the Kozak sequence are not the only nucleotides required for efficient binding and that a specific interaction between La and the Bcl2 mRNA occurs. Of note, compared to all three Bcl2_RNA oligonucleotides tested (Bcl2_WT, Bcl2_AUG, Bcl2_Kozak) computer-based prediction suggests that the two unrelated control_RNA oligonucleotides are not folding into a stable secondary structure (Supplementary Figure S4D and S4E). Taken together, we established specific binding of La to the Bcl2 mRNA (nucleotides −24 to +15), and that nucleotides surrounding the Bcl2 translation start site (Kozak), the start site itself as well as additional sequence/structural features are required for efficient binding of La to Bcl2 mRNA.

### The RNA chaperone activity of La restructures Bcl2 mRNA sequence surrounding the translation start site

**Figure 5:**
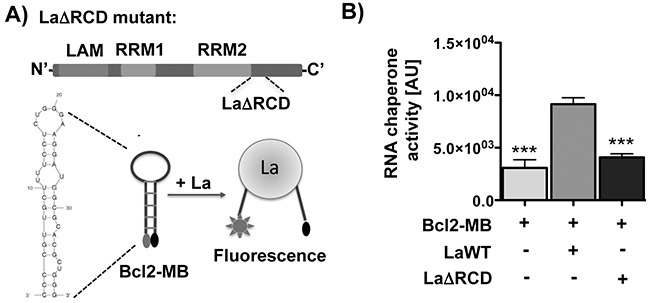
The RNA chaperone La assists structural changes of the Bcl2 translation start site *in vitro* **A.** The RNA chaperone domain (RCD) of La is mutated in LaΔRCD [21). RNA-binding motifs: LAM, RRM1, and RRM2. Scheme representing the RNA chaperone assay and the predicted structure of the Bcl2 RNA used as molecular beacon (Bcl2-MB). **B.** Differences in RNA chaperone activity given in relative fluorescence units (arbitrary units = AU) in the presence of 300 nM La wildtype protein (LaWT) compared to the molecular beacon (Bcl2-MB) alone or in presence of 300 nM recombinant La protein with mutated RNA chaperone domain (LaΔRCD). *P* value < 0.001 (three asterisks), n = 6.

Previous work suggests that the RNA chaperone activity of La is important to facilitate translation of cellular target mRNAs by assisting structural changes in 5′UTR region [1, 21]. Computer-based prediction of the Bcl2 mRNA region (nucleotides −24 to +15) including and surrounding the translation start site indicates a stable stem-loop structure. We reasoned that La binds to this region and destabilizes the stem due to its RNA chaperone activity. To test this notion we applied an RNA chaperone assay [21) and designed a molecular beacon (Bcl2-MB) spanning nucleotide −24 to +15 of Bcl2 RNA (Figure 5A). The Bcl2-MB was labeled at the 5′-end with a fluorophore (FAM fluorescein) and at the 3′-end with a quencher (Dabcyl). In closed stem-loop conformation the quencher would localize in close proximity to the fluorophore and reduce light emission after excitation whereas La-assisted helix-destabilization causes a spatial separation of the fluorophore from the quencher as measured by an increase in fluorescence light emission. Interestingly, our data show that La wildtype (LaWT), but not the RNA chaperone activity-deficient La mutant (LaΔRCD [21], Figure 5A) destabilizes the helical region within the Bcl2-MB resulting in increased fluorescence light emission (Figure 5B). In the LaΔRCD mutant basic amino acids are mutated within the carboxy-terminal region, which are required for the helix-destabilization activity of La [21]. Taken together, these data show that the RNA chaperone activity of La can restructure a stem-loop structured RNA molecule embedding the Bcl2 translation start site.

### RNA chaperone activity of La stimulates Bcl2 5′UTR-driven expression

**Figure 6:**
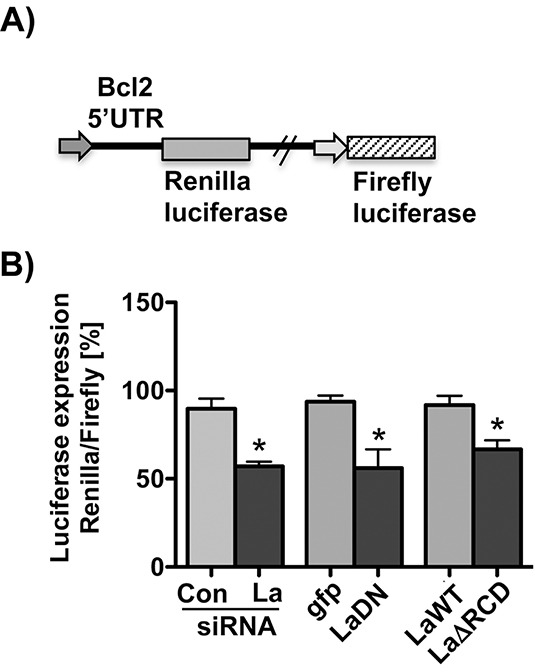
La-dependent Bcl2 5′UTR-driven reporter **A.** Scheme of the Bcl2 5′UTR-driven reporter in which the Bcl2 translation start site and surrounding sequences (nucleotides −286 to +29) were cloned upstream and in frame with the Renilla luciferase open reading frame. **B.** SiRNA-mediated La depletion or transient expression of La mutants (LaDN or LaΔRCD) reduces Renilla luciferase reporter expression in SCC 22B cells. *P* value < 0.05 (one asterisks), n = 3.

To test whether La is required to stimulate a luciferase reporter driven by the Bcl2 5′UTR, we cloned the Bcl2 translation start site and surrounding sequences (nucleotides −286 to +29) upstream and in frame with the Renilla luciferase open reading frame (Bcl2 AUG-driven Renilla luciferase reporter, Figure 6A). This reporter expresses independently Firefly luciferase used for normalization. Our data demonstrate that depletion of La by siRNA-mediated knockdown (La siRNA) or overexpression of dominant negative La mutant (LaDN) reduces Bcl2 AUG-driven Renilla luciferase expression compared to control siRNA (Con siRNA) or gfp-transfected cells, respectively (Figure 6B). To test whether the RNA chaperone domain of La contributes to Bcl2 start site-driven expression, we co-transfected the Bcl2 AUG-driven Renilla luciferase reporter with LaΔRCD mutant or La wildtype (LaWT) expression plasmid. The protein expression of both constructs was verified by immunoblot analysis (Supplementary Figure S3B). Comparing Renilla luciferase expression between LaWT- and LaΔRCD-expressing SCC 22B cells, we measured significantly less Renilla luciferase activity in LaΔRCD-expressing SCC 22B cells (Figure 6B), suggesting that the RNA chaperone activity of La stimulates Bcl2 5′UTR-driven expression.

### La stimulates Bcl2 mRNA translation in SCC 22B cells during cisplatin treatment

**Figure 7:**
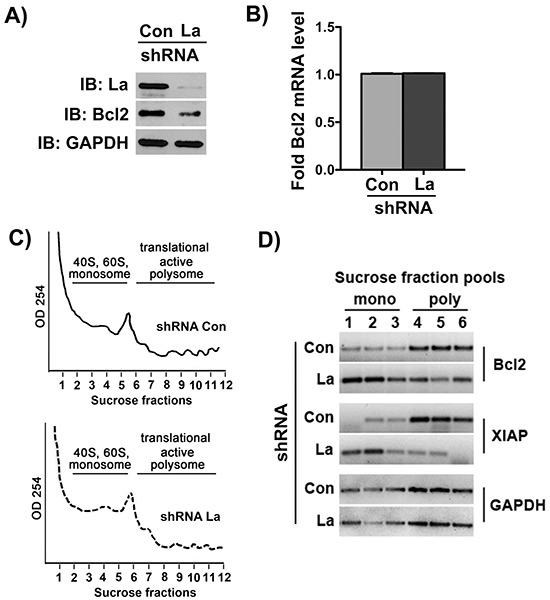
The La protein stimulates Bcl2 mRNA translation Cell lysates for polyribosome fractionation prepared from La-depleted (shRNA La) and control-treated (shRNA Con) SCC 22B cells treated with 24 μM cisplatin for 24 hours demonstrate that **A.** Bcl2 protein expression is reduced, but **B.** Bcl2 mRNA level normalized to GAPDH mRNA are unchanged. IB: immunoblot GAPDH expression was not affected and served as loading control. **C.** Representative polyribosome fractionation of two independent experiments of control-treated and La-depleted SCC 22B cells. **D.** For PCR amplification we pooled two fractions of the gradient, ending up with 3 samples representing the translational inactive portion of the gradient (mono) and three fractions of the translational active portion (poly) of the gradient. Agarose gel electrophoresis of RT-PCR products amplified from Bcl2, XIAP and GAPDH mRNA isolated from monosome (mono) and polysome (poly) fractions demonstrates that Bcl2 and XIAP mRNA accumulates in translational inactive fractions in La-depleted SCC 22B cells. The translation of GAPDH mRNA is not affected by La depletion.

To ascertain that La stimulates Bcl2 mRNA translation during cisplatin treatment, we performed polyribosome fractionation and analysis on control-treated and La-depleted SCC 22B cells. Therefore, stably transduced shCon- and shLa-RNA expressing SCC 22B cells were treated with 24 μM cisplatin for 24 hours, and protein as well as total RNA level of Bcl2 were checked by immunoblot analysis and RT-qPCR, respectively (Figures 7A and 7B). Cytosolic cell extracts from those experimental settings were fractionated by sucrose gradient centrifugation (Figure 7C). Total RNA from each sucrose gradient fraction was prepared, assessed for integrity, and pooled RNA fractions (two fractions each) were analyzed by RT-PCR for Bcl2. Strikingly, our results showed a strong shift of Bcl2 mRNA from polysome fractions (translational active) in control-treated cells to monosome fractions (translational inactive) in La-depleted cells, demonstrating that La is required for active Bcl2 mRNA translation in SCC 22B cells (Figure 7D). Furthermore, we also monitored the distribution of XIAP mRNA in the gradient and observed a shift of XIAP mRNA from translational active to translational inactive fractions in La-depleted cells (Figure 7D) as expected, since it has been previously published that translation of IRES-containing XIAP mRNA is La-dependent [12]. In contrast, GAPDH mRNA translation was not affected by changes in La protein levels (Figure 7D). These studies establish that the La protein stimulates Bcl2 mRNA translation in SCC 22B cells during cisplatin treatment. Detailed studies of the mechanism how La mutants, like LaDN or LaΔRCD, Bcl2 mRNA translation are part of an ongoing study.

## DISCUSSION

For the first time we present data that the overexpression of RNA-binding protein La protects against cisplatin-induced cell death and that depletion of the La protein sensitizes HNSCC cells to cisplatin. These findings are significant because the La protein is overexpressed in various types of cancer and this study suggests that high La levels contribute to cancer pathogenesis and chemotherapy resistance by counteracting therapeutic treatments aiming to induce cell death. Exploring the underlying mechanism, we demonstrate *in vitro* that La binds in close proximity to the translation start site of Bcl2 mRNA and is able to open a stem-loop structure embedding the translation start site due to its RNA chaperone activity. In cell-based assays we demonstrated the existence of an endogenous La:Bcl2 mRNA complex and established that La stimulates protein synthesis of anti-apoptotic factor Bcl2.

Cisplatin is a widely used platinum-based compound approved as an anticancer drug to treat a wide spectrum of advanced cancers that cannot be treated with surgery or radiation therapy including head and neck squamous cell carcinoma, cervical and lung cancer. A major obstacle for cancer patients during chemotherapeutic treatment is intrinsic or acquired drug resistance of cancer cells [24, 25]. Hence we need to uncover, characterize, and finally target molecular mechanisms of drug resistance to ensure more efficient anti-cancer treatment in the future [40]. Chemoresistance of cancer cells to cisplatin treatment typically has a multifactorial nature including reduced drug uptake, increased drug inactivation, increased DNA adduct repair, or overexpression of anti-apoptotic factors counteracting the therapeutic stimulus for cell death. Therefore, inhibitors of anti-apoptotic factors like Bcl2, XIAP and Survivin were tested in clinical trials [24, 26]. Interestingly, also the La-related protein 1 (LARP1) has recently been found to promote survival and cisplatin resistance by stabilizing Bcl2 mRNA in in ovarian cancer cells [41].

Overexpression of anti-apoptotic factor Bcl2 in cancer cells can be achieved by various post-transcriptional mechanisms, for example aberrant expression of miRNAs [42] or increased protein phosphorylation and stabilization resulting in resistance toward cisplatin-induced cell death [43]. Here, we show that the La protein stimulates Bcl2 expression by binding to its mRNA and we propose that targeting the La:Bcl2 mRNA interaction might represent a novel therapeutic approach to reduce Bcl2 protein synthesis.

Although the molecular mechanism whereby La contributes to proteins synthesis is still not well understood, it is very likely that La has to bind to the target mRNA. Hence blocking the binding of La would be expected to impair target mRNA expression. Based on earlier studies, La might support correct translation start site usage during initiation of protein synthesis [15]. One interesting aspect, which is supported by this study, is that La stimulates translation of mRNAs in which the translation start site is embedded in a stem-loop structure as suggested for HCV [10, 17, 19, 20, 38] and cyclin D1 [2, 21]. As shown herein and described earlier [39], La prefers binding to translation start sites embedded in a Kozak sequence, although our data show that this is not sufficient because competitor control RNA with a strong Kozak-embedded AUG cannot compete in binding assays. The observation that the non-functional competitor control RNA is not folding into a stable stem-loop structure is suggesting that La favors binding to strong AUGs embedded in a structural context. In our model translation start sites buried in strong stem-loop structures might not be efficiently recognized by the scanning 43S ribosomal subunit until the RNA chaperone La binds and opens the structure and exposes the translation start site.

Herein we present that La stimulates mRNA translation of anti-apoptotic factor Bcl2 in cisplatin-treated HNSCC cells. These findings may have significant impact on chemotherapeutic treatment since La is not only overexpressed in HNSCC [3], but also in various other types of cancer like chronic myeloid leukemia [1], cervical cancer [2], as well as lung, prostate and breast tumor tissue (unpublished data). Our model implies that cancer-associated La facilitates mRNA translation of anti-apoptotic factors, offsets cisplatin-induced cell death and thereby contributes to cisplatin resistance of cancer cells. This scenario suggests that developing molecular drugs inhibiting the binding of La to mRNAs encoding anti-apoptotic factors may sensitize cancer cells toward cisplatin treatment. Taken together, we identified a novel regulatory mechanism contributing to cisplatin resistance and suggesting that targeting this mechanism might establish a highly innovative and promising anti-cancer strategy.

## MATERIALS AND METHODS

### Cell culture, siRNA transfection and shRNA lentiviral transduction

SCC 4, SCC 25, U2OS, HEK 293 and NIH 3T3 cells were purchased from ATCC. Cell lines UM-SCC 22A (SCC 22A) and UM-SCC 22B (SCC 22B) were described elsewhere [3, 44]. SCC 4 and SCC 25 cells were cultured in DMEM:Ham F12 (Hyclone) containing 400 ng/ml Hydrocortisone (Sigma) plus 10% FBS (Atlanta Biologicals). SCC 22A, SCC 22B, U2OS, HEK 293 and NIH 3T3 cells were cultured in advanced DMEM (Gibco) containing 2mM L-glutamine (Life Technology) and 10% FBS. All cell lines were tested for mycoplasma contamination by applying MycoSensor PCR Assay kit according to manufactur's instructions (Agilent Technologies).

La-specific siRNA-mediated depletion and rescue experiments were performed as described recently [2, 3]. Transduction of MISSION shRNA constructs significantly reduced La expression (TRCN0000062193 and TRCN0000062195) (> 80%) and were used for all shRNA lentiviral transduction experiments according to the manufacture's instruction (Sigma).

### Annexin V staining and cisplatin IC_50_ determination

Apoptotic cells were determined with the Annexin-V-FLUOS Staining Kit (Roche) according to the manufacturer's instruction. The half maximal inhibitory concentration (IC_50_) was determined as response to cisplatin by treating 2×10^4^ cells (SCC 22A, SCC 25) or 1×10^4^ cells (SCC 22B, SCC 4) with increasing cisplatin (Selleckchem) concentrations or vehicle (DMSO) for 48h (96-well format, Supplementary Figure S1). Cells were washed twice with 1x PBS and quantified by staining with fluorescence dye (CyQUANT®, Life Technologies). The IC_50_ values were calculated applying Prism 5 (GraphPad Software).

### Plasmids and cloning

Cloning of gfp-tagged dominant-negative La mutant (LaDN = aa 226-348 [33]) was performed by PCR using oligonucleotides LaDNs/LaDNas, and gfp-tagged La plasmid [45] as a template. The oligonucleotides used for cloning areLaDNs = 5′-ATGAATTCCGAGCTCTAGAAGAAAAGATTGGATGCTTG and LaDNas = 5′-ATGATATCAAGCTTCTAAGGCTGGGCAGCTTTATTACC. The PCR product was cloned via *Sac*I and *Hind* III into vector pEGFP-C1 (Clonetech). Cloning of gfp-tagged La wildtype (LaWT) and siRNA resistant (LaR) La mutant has been described earlier [2]. The ER-targeted Bcl2 (Bcl2^ER^) plasmid (Bcl2GFP-Bcl2-Cb5) [35] was purchased from Add gene (Reference). For cloning of the Bcl2 5′UTR reporter, PCR was performed using oligonucleotides B2_Pecks/as and Bcl2 cDNA isolated from SCC 22B as template. The PCR product (Bcl2 mRNA −286 to +29) was cloned via NheI into vector pCheck-2 (Promega). Orientation of the cloned DNA fragment was checked by KpnI digestion, which was introduced into the PCR fragment with the B2_Pecks PCR primer. B2_Pecks (KpnI restriction site underlined): GAATTCGCTAGCGGTACCCATCACAGAGGAAGTAGACTG, B2_Peckas: GCTTTTCCTCTGGGAAGGATGGCGCACGCTGGGAGAACAGGGCTAGCGATATC.

All plasmids were sequenced for verification and to confirm mutagenesis.

### Total RNA isolation, RT-qPCR and standard PCR

Total RNA was isolated (TriPure, Roche), genomic DNA was digested with TURBO™ DNase(Ambion), and RNA was reverse transcribed (RT) applying the ThermoScript RT–PCR System (Invitrogen) or RT^2^ First Strand Synthesis Kit (Qiagen). Quantitative PCR (qPCR) was performed on the BioRad iCycler-iQ (BioRad) using the RT^2^ SYBR Green Fluor qPCR Mastermix (Qiagen) and the QuantiTect Primer Assays (Qiagen): Hs_Bcl2_vb.1_SG, or RT^2^ qPCR Primer Assays (Qiagen): Bcl2 (PPH00079B), GAPDH (PPH00150F), XIAP (QT00042854), hnRNPE2 (QT00091427), and CCNA2 (QT00014798). Quantitative PCR was performed by relative standard curve method, where the standard plot was constructed by using known concentration of template for each primer set. Standard PCR was performed with iProof™ High-Fidelity DNA Polymerase (BioRad) using the following primer: GAPDH sense 5′-ACCACAGTCCATGCCATCAC; GAPDH antisense 5′-TCCACCACCCTGTTGCTGTA; Bcl2 sense (B2P1T7, see below); Bcl2 antisense (B2P1as, see below); XIAP sense: 5′-GAGAATTCACTAGTATTAGAATGTTTCTTAGCGGTCG; XIAP antisense: 5′-ATGATATCCCATGGCTTCTCTTGAAAATAGGACTTGTCCACCTTTTCTAAAAAGAG.

### RNA pull-down down assay

For RNA pull-down assays we followed a protocol provided by Myriam Gorospe (National Institute on Aging) [46]. PCR primer combinations were used to generate templates for *in vitro* transcription to create biotinylated RNA probes: FL (full length) template = B2P1T7/B2P2as spanning −286 to +3, P1 template = B2P1T7/B2P1as spanning −286 to −126, and P2 template = B2P2T7/B2P2as spanning −125 to +3. Oligonucleotide sequences (T7 promoter sequence is underlined):B2P1T7: 5′-GAATTCAAGCTTAATACGACTCACTATAGGGCATCACAGAGGAAGTAGACG; B2P1as: 5′-GATATCGGATCCACGAGGGGGTGTCTTCAATC; B2P2T7: 5′-GAATTCAAGCTTAATACGACTCACTATAGGGCCAAGAATGCAAAGCACATCC; B2P2as: 5′-GATATCGGATCCCATCCTTCCCAGAGGAAAAGC.

### RNA immunoprecipitation (RIP)

RIP experiments in SCC 22B, U2OS and NIH 3T3 cells for endogenous Bcl2 were performed by *in vivo* crosslinking and immunoprecipitation of La-RNA complexes applying the monoclonal anti-La SW5 [47, 48]. anti-body or purified mouse immunoglobulin isotype IgG 2b,k (eBioscience) and has been described earlier [2]. For RIP experiments in HEK 293 cells we first generated stable cell lines (G418 selection) expressing gfp or gfp-tagged La. RIP experiments of HEK 293 cells stably overexpressing gfp or gfp-tagged La were performed by harvesting cells at 60-80% confluence. Cells were washed with ice-cold PBS and lysed with lysis buffer (20 mM Tris-HCl, pH 7.4, 150 mM NaCl, 1% IGEPAL CA-630, 10% glycerol, 1 mM EDTA, 50mM NaF, and 1 mM DTT) supplemented with RNase inhibitors and protease inhibitors. An aliquot of the cleared lysate was used directly for RNA preparation (Input). The cleared lysate was incubated with GFP-magnetic beads (MBL International) overnight on the orbital rotor at 4°C. The beads were washed four times with wash buffer I (50 mM Tris-HCl, pH 7.4, 300 mM NaCl, 0.05 % IGEPAL CA-630, 20 mM EDTA, 1 mM DTT, and 1 mM MgCl_2_) and three times with wash buffer II (50 mM Tris-HCl, pH 7.4, 300 mM NaCl, 0.05 % IGEPAL CA-630, 20 mM EDTA, 1 mM DTT, 1 mM MgCl_2_ and 1 M urea). The RNA was isolated from the RIP pellet (pellet) and subjected to RT-qPCR analysis. The enrichment of a specific RNA in the RIP pellet was determined by calculating the percentage of the respective mRNA in the pellet versus the input material. The relative enrichment was calculated by using the formula: enrichment of target mRNA/enrichment of GAPDH mRNA. Control experiments were performed with gfp-expressing cells to assess the background.

### Non-radioactive electrophoretic mobility shift assay (EMSA) and fluorescence polarization (FP) assay

The binding affinity of wildtype recombinant La protein to the Bcl2_WT RNA was determined by native EMSA and FP assay, as described recently [21]. The Bcl2_WT RNA oligonucleotide was synthesized and 5′-end labeled with the fluorophore 6-carboxyfluorescin (FAM) by Integrated DNA Technologies, Inc.: 5′-/56FAM/CCCGUUGCUUUUCCUCUGGGAAGGAUGGCGCACGCUGGG.

La binding curves were established at 25 nM Bcl2_WT RNA oligonucleotide and increasing concentrations (0, 20, 40, 60, 100, 200, 300, 400, 500, and 600 nM) of recombinant His-tagged La protein. Competitive FP assays were performed with 25 nM of Bcl2 RNA oligonucleotide, 200 nM of La protein and 4-, 10-, 40-, 80-fold competitor RNA oligonucleotide (Figure 4E, Supplmentary Figure S3A). The dissociation constants (K_D_) were determined using Prism 5 (GraphPad Software).

### RNA chaperone assay

The Bcl2 RNA molecular beacon Bcl2-MB was synthesized, 5′-end labeled with the fluorophore 6-carboxyfluorescin (FAM), and 3′end labeled with a quencher (Dabcyl) by Integrated DNA Technologies, Inc.: /56-FAM/CCCGUUGCUUUUCCUCUGGGAAGGAUGGCGCACGCUGGG/3Dab/. The RNA chaperone assays were performed with 25 nM Bcl2-MB and 300 nM La protein as described recently [21].

### Polyribosome fractionation

This technique has been described earlier [49]. For sucrose gradient centrifugation 100 μg/ml cycloheximide was added to culture medium for 5 min. Cells were washed, harvested in lysis buffer (15 mM Tris/HCl, pH 7.4, 15 mM MgCl_2_, 300 mM NaCl, 0.1% Triton X100, 200 units/ml RNAsin (Promega), 0.1% mercaptoethanol, 10 μg/ml cycloheximide, including protease inhibitors), incubated for 10 min at 4°C and centrifuged for 10 min at 4°C at 9,300xg. The supernatant was loaded onto sucrose gradients (17.5 to 50%) and ultra centrifuged. The gradients were fractionated and RNA was extracted using the phenol:chloroform:isamylalcohol method, measured and analyzed using an Bioanalyzer instrument.

### Protein lysates, protein purification and immunoblot analysis

Normal tongue tissue and HNSCC tissue lysates (T13-001, −002, −003, −004, −008, −009) were obtained from Protein Biotechnologies. Purification of the His-tagged recombinant La proteins has been described earlier [21]. For immunoblot analysis the following antibodies were used: anti-La 3B9 [2, 3, 47, 48], anti-GFP (Roche) and anti-GAPDH (FL-335) were purchased from Santa Cruz and anti-Bcl2 (D55G8) was purchased from Cell Signaling. Quantification of immunoblots was performed by recording of chemiluminescence signals using an ImageQuant ECL systems or BioRad Chemiluminescence Imager and quantified using ImageQuant TL or Biorad Chemiluminescence Imager software, respectively.

### Statistical considerations

Two-tailed *P value* was determined by t-test applying Prism 5 (GraphPad Software). *P* value < 0.05 (one asterisks), < 0.005 (two asterisks) and < 0.001 (three asterisks). Error bars represent the mean+/−SD in n-independent experiments.

## SUPPLEMENTARY FIGURES


