# Supplementary material for: The La protein counteracts cisplatin-induced cell death by stimulating protein synthesis of anti-apoptotic factor Bcl2
Source: Oncotarget. 2016 Apr 18;7(20):29664–76. doi: 10.18632/oncotarget.8819 (PMC5045424; doi:10.18632/oncotarget.8819)
Supplement: Supplementary file 1 [file oncotarget-07-29664-s001.pdf]

SUPPLEMENTARY FIGURES

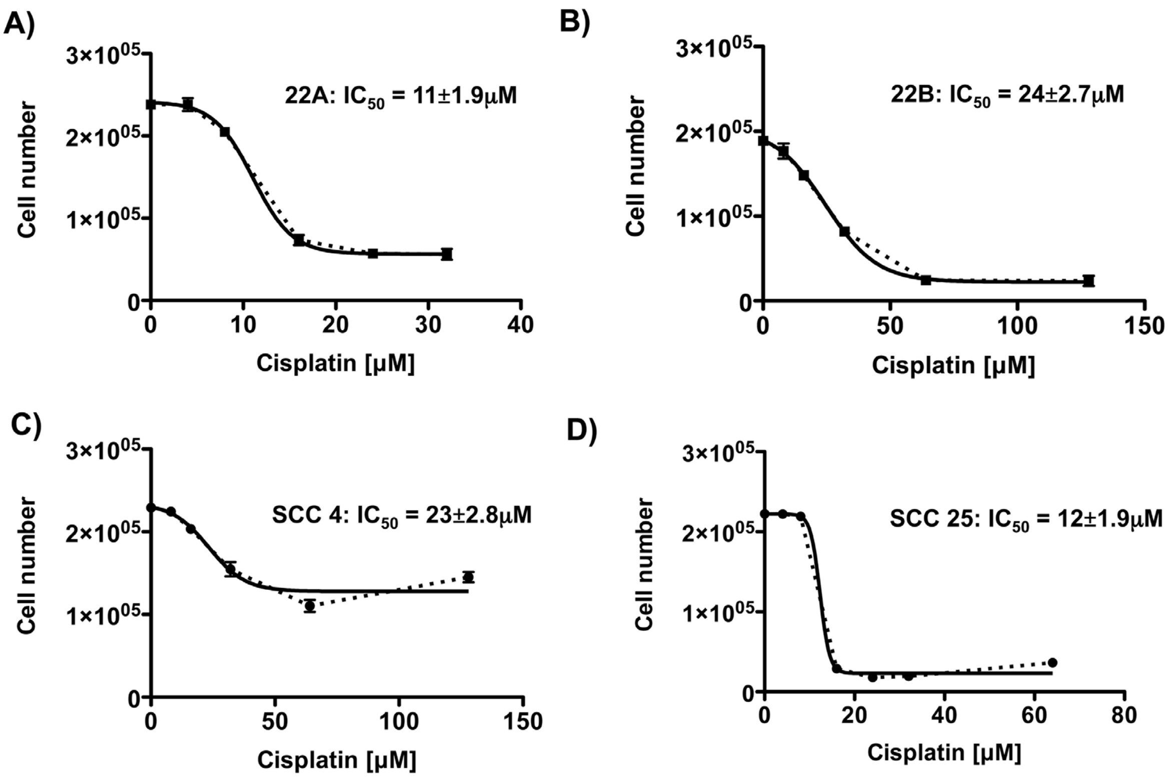

Supplementary Figure S1: Cisplatin IC_{50} of four different HNSCC cell lines. A. SCC 22A (IC_{50} = 11+/-1.9 µM), **B.** SCC 22B (IC_{50} = 24+/-2.7 µM), **C.** SCC 4 (IC_{50} = 23+/-2.8 µM), and **D.** SCC 25 (IC_{50} = 12+/-1.9 µM). At least two independent experiments were performed in triplicates. The IC_{50} values were calculated applying Prism 5 (GraphPad Software).

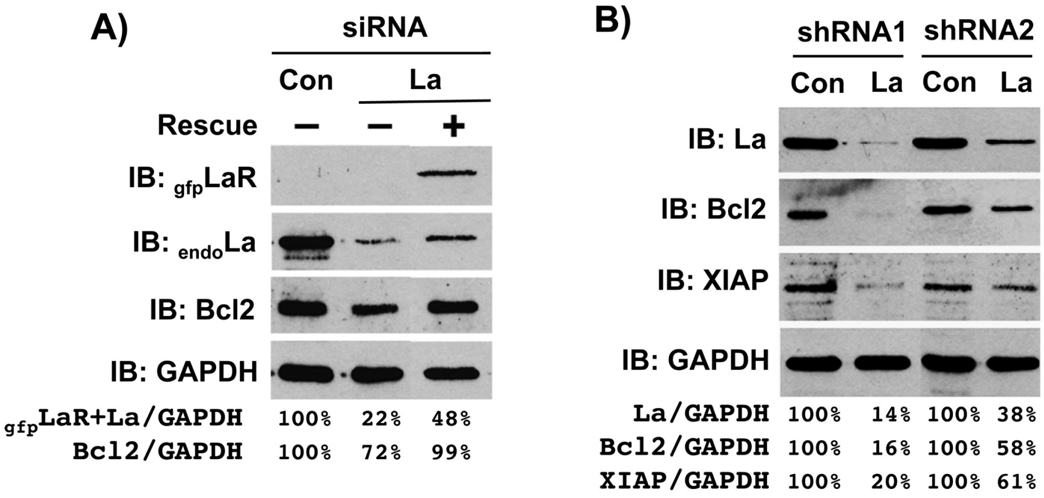

Supplementary Figure S2: A. Bcl2 protein expression can be rescued in La-depleted (La siRNA) SCC 22B cells by overexpressing gfp-tagged La resistant to La-specific siRNA ($_{gfp}$LaR). GAPDH was used as loading control. Con = control siRNA. **B.** Bcl2 and XIAP protein expression is reduced in SCC 22B cells transduced with two independent La-specific shRNAs. For quantification of immunoblot (IB) signals an ImageQuant ECL system and the ImageQuant TL software was used.

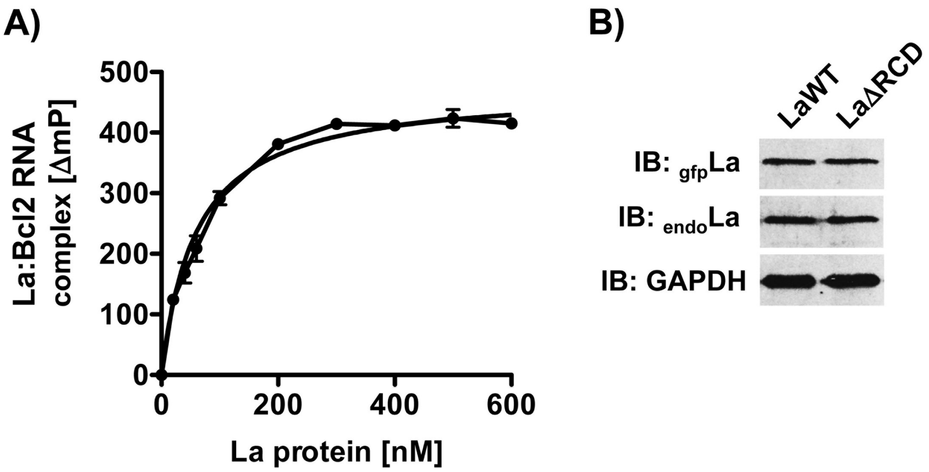

Supplementary Figure S3: A. Binding affinity for the La:Bcl2 RNA interaction as determined by fluorescence polarization assays. The La:RNA complex formation as difference in polarization (ΔmP) was plotted against the La protein concentration. The K_D was determined as 60.1+/-4.5 nM (n=6) in Prism 5 (GraphPad Software). **B.** immunoblot (IB) analysis to validate equal expression of La wildtype (LaWT) and La RNA chaperone domain (LaDRCD) mutant in SCC 22B cells.

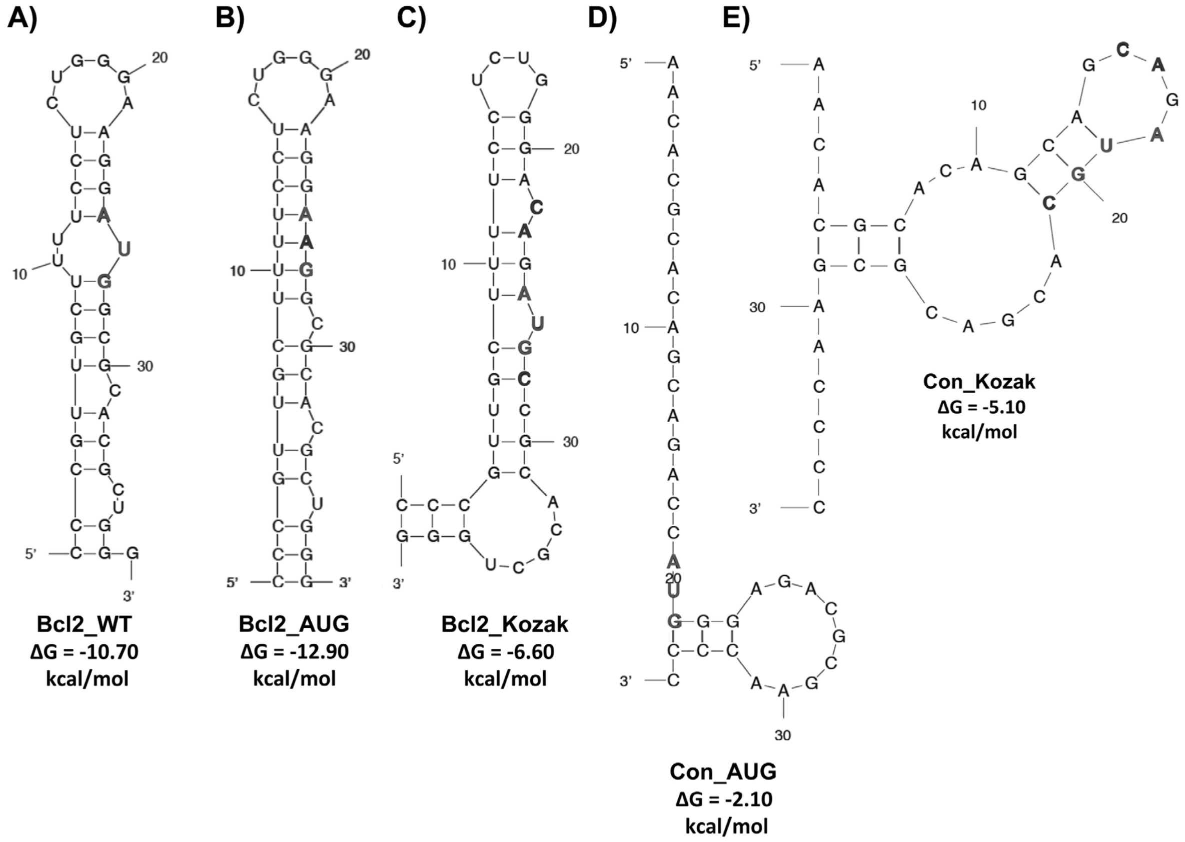

Supplementary Figure S4: Secondary structure of Bcl2 RNA oligonucleotides and two unrelated control (Con) RNA oligonucleotides as determined by applying the RNA Folding Form available on the mfold Web Server (http://mfold.rna.albany.edu/?q=mfold, The RNA Institute, Albany, USA) [50]. **A.** Bcl2_WT, **B.** Bcl2_AUG, **C.** Bcl2_Kozak, **D.** Con_AUG, and **E.** Con_Kozak.
